# Integrating Ethnobotany, Phytochemistry, and Pharmacology of *Cotinus coggygria* and *Toxicodendron vernicifluum*: What Predictions can be Made for the European Smoketree?

**DOI:** 10.3389/fphar.2021.662852

**Published:** 2021-04-19

**Authors:** Diana Simona Antal, Florina Ardelean, Robert Jijie, Iulia Pinzaru, Codruta Soica, Cristina Dehelean

**Affiliations:** ^1^ Department of Pharmaceutical Botany, Faculty of Pharmacy, “Victor Babes” University of Medicine and Pharmacy, Timisoara, Romania; ^2^ Department of Toxicology, Faculty of Pharmacy, “Victor Babes” University of Medicine and Pharmacy, Timisoara, Romania; ^3^ Department of Pharmaceutical Chemistry, Faculty of Pharmacy, “Victor Babes” University of Medicine and Pharmacy, Timisoara, Romania

**Keywords:** *Cotinus coggygria*, *Toxicodendron vernicifluum*, common metabolites, sulfuretin, fisetin

## Abstract

The smoketree (*Cotinus coggygria*) is a historically known medicinal plant from Southeast Europe. Its ethnomedicinal use in skin and mucosal lesions is commonly accepted across countries. Other utilizations reported locally include fever reduction, cardiac diseases, hypertension, urinary diseases, cough, asthma, hemorrhoids, diabetes, numbness of arm, liver disease, and cancer. Departing from the smoketree’s traditional uses, this review summarizes investigations on the phytochemistry and bioactivity of the plant. *In vitro* and *in vivo* experiments supporting wound-healing, anti-inflammatory, antibacterial, cytotoxic, antioxidative, hepatoprotective, and antidiabetic effects are presented. Metabolites from smoketree that are responsible for the main pharmacological effects of smoketree are pointed out. Furthermore, the review performs a comparison between *C. coggygria* and the lacquer tree (*Toxicodendron vernicifluum*). The latter is a comprehensively studied species used in Asian phytotherapy, with whom the European smoketree shares a consistent pool of secondary metabolites. The comparative approach aims to open new perspectives in the research of smoketree and anticipates an optimized use of C. coggygria in therapy. It also points out the relevance of a chemosystematic approach in the field of medicinal plants research.

## Introduction

Smoketree (*Cotinus coggygria* Scop., syn. *Rhus cotinus* L.) is a shrub growing wildly on sunny limestone slopes in the Balkans, Southern and Eastern Europe, the Caucasus and Central Asia (Matić et al., 2016). Aside from its natural occurrence, smoketree cultivars with colorful foliage are frequently planted in parks and gardens. Smoketree wood, distinctive through its yellow color, has been employed as a dye since ancient times. Key chemical constituents of the golden wood have been revealed in historical textiles from different parts of Europe and Asia: liturgical garments from the Athos Mountain (Valianou et al., 2009; Mantzouris et al., 2011), religious embroideries, brocaded velvets and other ethnographical fabrics from Romania (Petroviciu et al., 2014), even Chinese textiles from Dunhuang, dating back to over thousand years (Tamburini, 2019). Aside from its importance as a dye, smoketree was used for therapeutic purposes since Antiquity. The use of the name “*Cotinus coccigria*” dates back to Theophrastus (1644) according to the treatize De Historia Plantarum, one of the most important books on the structure and use of plants. However, Linnaeus had first included the smoketree in the *Rhus* genus, under the name *Rhus cotinus* (Linnaeus, 1753). The currently used name, *Cotinus coggygria*, was conferred by (Scopoli, 1772). The genus name, “*Cotinus*”, means “wild olive” and is of Greek origin (“κoτινος”), while *coggygria* stems from the Greek “κοκκυγέα” meaning smoke tree.

The knowledge of the therapeutic qualities that has been passed on from generation to generation has lead to a reservoir of information that became the subject of modern research aimed at validating these effects through *in vitro* and *in vivo* experiments. Interestingly, a closely related species, previously classified in the same genus as the European smoketree, *Rhus*, shares a significant amount of common constituents. This species, called Asian lacquer (*Toxicodendron vernicifluum* or *Rhus verniciflua*) proved to have many similar indications, but also new ones. By integrating the phytochemistry and pharmacology of these two species, the current review intends to advance and expand the practical use of the European species which has the advantage of being free of allergenic urushiols, as opposed to its Asian counterpart (Ippen, 1983; Kim et al., 2014).

## Taxonomy and Botanical Aspects of *Cotinus coggygria*

The plant is a member of the Anacardiaceae family, which includes tropical representatives like mango (*Mangifera indica*) and cashew (*Anacardium occidentale*), but also species vegetating under temperate climates, for example the allergenic poison sumac (*Rhus toxicodendron*). *C. coggygria* is multi-stemmed, deciduous, averaging heights of 5–7 m. Its leaves have an ovate or obovate shape, pinnate venation and a glaucous lower face. The small flowers are grouped in large panicles, with the pedicels elongating into hairy stalks that cover the inflorescences with smoke-like puffs. This “smoked” aspect inspired several names of the plant: “smoketree”, “smoke bush”, and even “wig tree”. The species is one of the seven of the genus *Cotinus* Mill., the others having a narrower distribution range, in North America: *C. obovatus* (native to Central and South East of the United States), *C. carranzae* and *C. chiangii* (both native to Mexico), and in Asia: *C. kanaka* (native to the southern part of the Eastern Himalayas), *C. nanus* and *C. szechuanensis* (growing in South-Central China)^1^.

## Traditional Uses

*C. coggygria* has a consistent traditional use in Europe and Asia. The Encyclopedia of Romanian Ethnobotany points to the use of the plant as both a dye and medicine, employed for the treatment of wounds and pharyngitis (Butură, 1979). An enthnobotanical survey performed in the region of Dobrogea (South-East Romania) reported on the use of the plant as a cicatrizing agent of open wounds and the treatment of gynecological disorders (Tudor and Georgescu, 2011). The application of *C. coggygria* (called “skumpina”) has also been acknowledged in a survey focused on plants used by the Czech diaspora from Romanian Banat region (Vlková et al., 2015). In the local folk medicine of Southwestern Romania, several parts of the smoketree are used: young twigs and leaves boiled in water represent a remedy against sore throat, stomatitis, gingivitis and gastritis. More conspicuously, wood slices extracted in badger fat (*Meles*) were mentioned as ointment ingredients intended to treat slowly healing wounds (Antal and Ardelean, 2015). The ethnomedical use in skin and mucosal lesions is commonly accepted across countries, according to reports from Bulgaria (Nedelcheva, 2012; Koleva et al., 2015), Bosnia and Herzegovina (Redžić, 2007), Serbia (Jarić et al., 2015), Albania (Nedelcheva and Draganov 2014), and Turkey (Kültür, 2007). Other uses include fever reduction (Huang and Williams 1998; Redžić, 2007), cardiac diseases, hypertension, urinary diseases, cough, asthma, hemorrhoids, diabetes, numbness of arm (Kültür, 2007), and liver disease (Shen et al., 1991). The plant is as well mentioned in cancer treatment in Turkey (Kültür, 2007) and Bosnia and Herzegovina (Redžić, 2007). Usually, leaves are the most frequently used plant parts in traditional medicine (as infusions or fresh).

## Phytochemistry of *Cotinus coggygria*

Smoketree is characterized by a broad spectrum of polyphenolic secondary metabolites: tannins, various subtypes of flavonoids, phenolic acids, as well as by the presence of volatile organic compounds. Essential oil components are contained in secretory ducts associated with the phloem; ducts run through the cortex of twigs as well as the petiole and midrib of leaves (Metivier et al., 2007; Antal and Ardelean, 2015; Özcan and Yilmaz, 2020).

### Tannins

**FIGURE 1:**
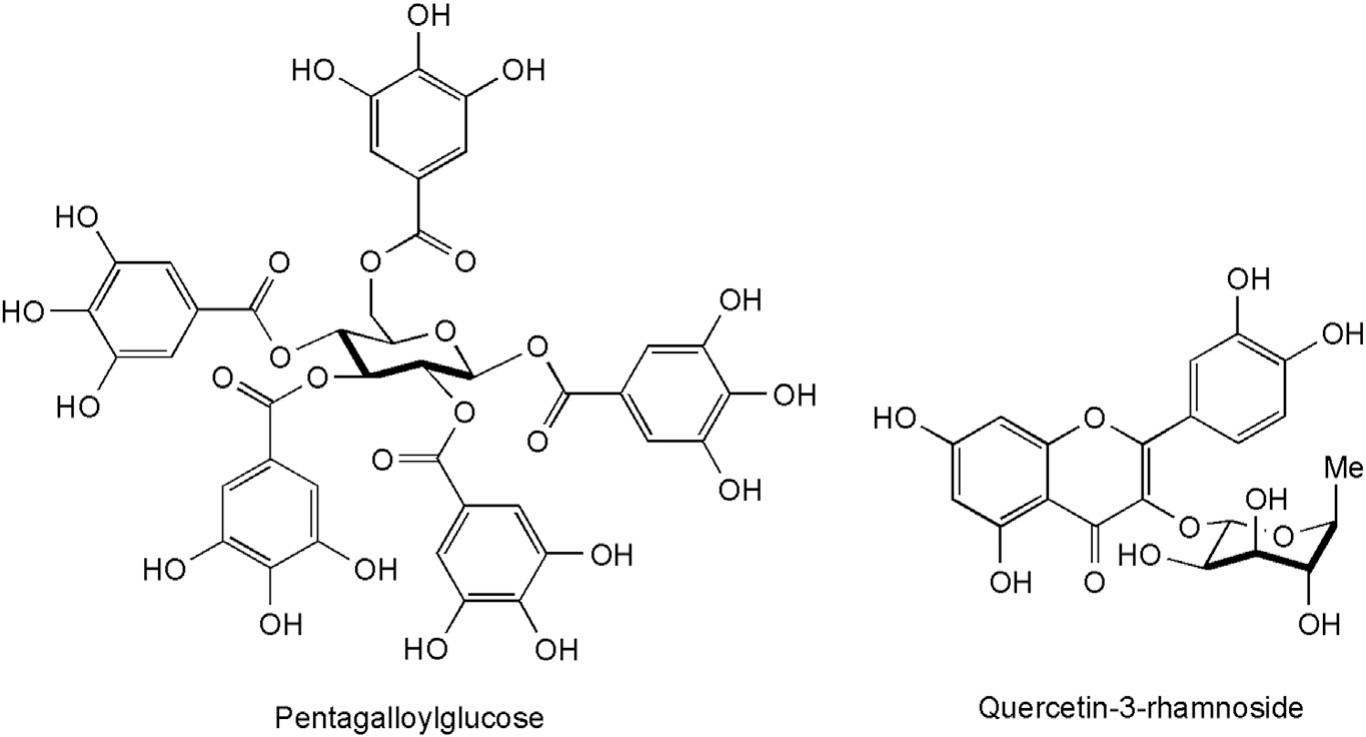
Representative compounds from the leaves of *C. coggygria*.

These constituents are abundantly present in *C. coggygria*, a fact that relates to its taxonomic affiliation to the Anacardiaceae family–a group with economically important tanning plants like quebracho (*Schinopsis* spp.) and sumacs (*Rhus* spp.). These polyphenols are able to establish bonds with proteins, and precipitate them–a property which is at the heart of their bioactivity. Their interaction with dermal collagen fibers explains their cicatrizing effects, while the precipitation of bacterial proteins by tannins correlates with their antibacterial effect (Bruneton, 2016). Smoketree contains significant amounts of hydroysable tannins derived from gallic acid. Pentagalloyl glucose (1,2,3,4,6-penta-O-galloyl-β-D-glucopyranose, Figure 1), the ester of glucose with five moieties of gallic acid has been one of the first tannins identified from the plant (Westenburg et al., 2000). The tannin content of leaves was reported to be highest during the flowering period, attaining 18–20% both according to earlier (Buziashvili et al., 1973) and more recent research (Šavikin et al., 2009). The most extensive data on the chemical structure of leaf tannins were reported by Rendeková and co-workers; identifications were performed upon comparison of UV and mass spectra with those from the literature or authentic standards. The presence of galloyl glucose (glucogallin), protocatechuic acid hexoside, trigalloyl hexoside, and tetragalloyl hexoside is accordingly added to the previously known hydrolysable tannins (Rendeková et al., 2016). Gallocatechin was confirmed in the leaves by (Özbek et al., 2016).

### Flavonoids

Among plant species, smoketree stands out through the presence of nearly all flavonoid subclasses. These compounds have especially been investigated in stems and branches, as these parts are used for the obtainment of an orange-hued dye, containing a mixture of flavonoids.

### The Heartwood

**FIGURE 2:**
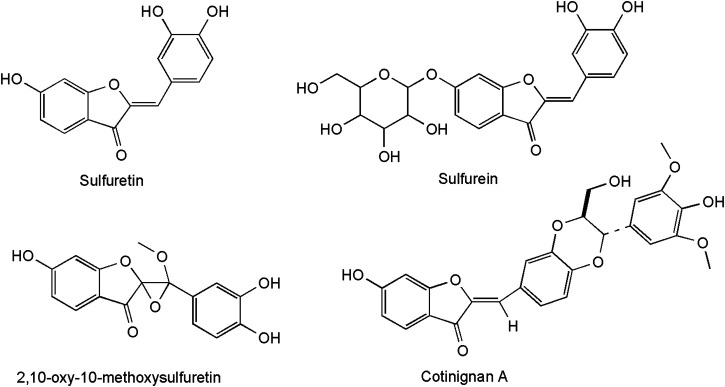
Structure of condensed tannins from *C. coggygria*.

The most representative metabolite class of *C. coggygria* in the heartwood are the aurones: sulfuretin (3′,4′,6-trihydroxyaurone), its glucoside sulfurein (glycosil-7-O-sulfuretin), and its dimer disulfuretin (Westenburg et al., 2000). The latest discovery concerns two new metabolites from the plant kingdom: an aurone epoxide (2,10-oxy-10-methoxysulfuretin) and an auronolignan (Figure 2). In cotinignan A, the first compound of this class, the aurone sulfuretin is bridged to sinapyl alcohol by a benzodioxane ring (Novaković et al., 2019).

Other orange-colored compounds from smoketree wood are butein (trans-2′,3,4,4′-tetrahydroxychalcone) and isoliquiritigenin (trans-2′,4,4′-trihydroxychalcone), members of the chalcone subclass of flavonoids (Valianou et al., 2009).

**FIGURE 3:**
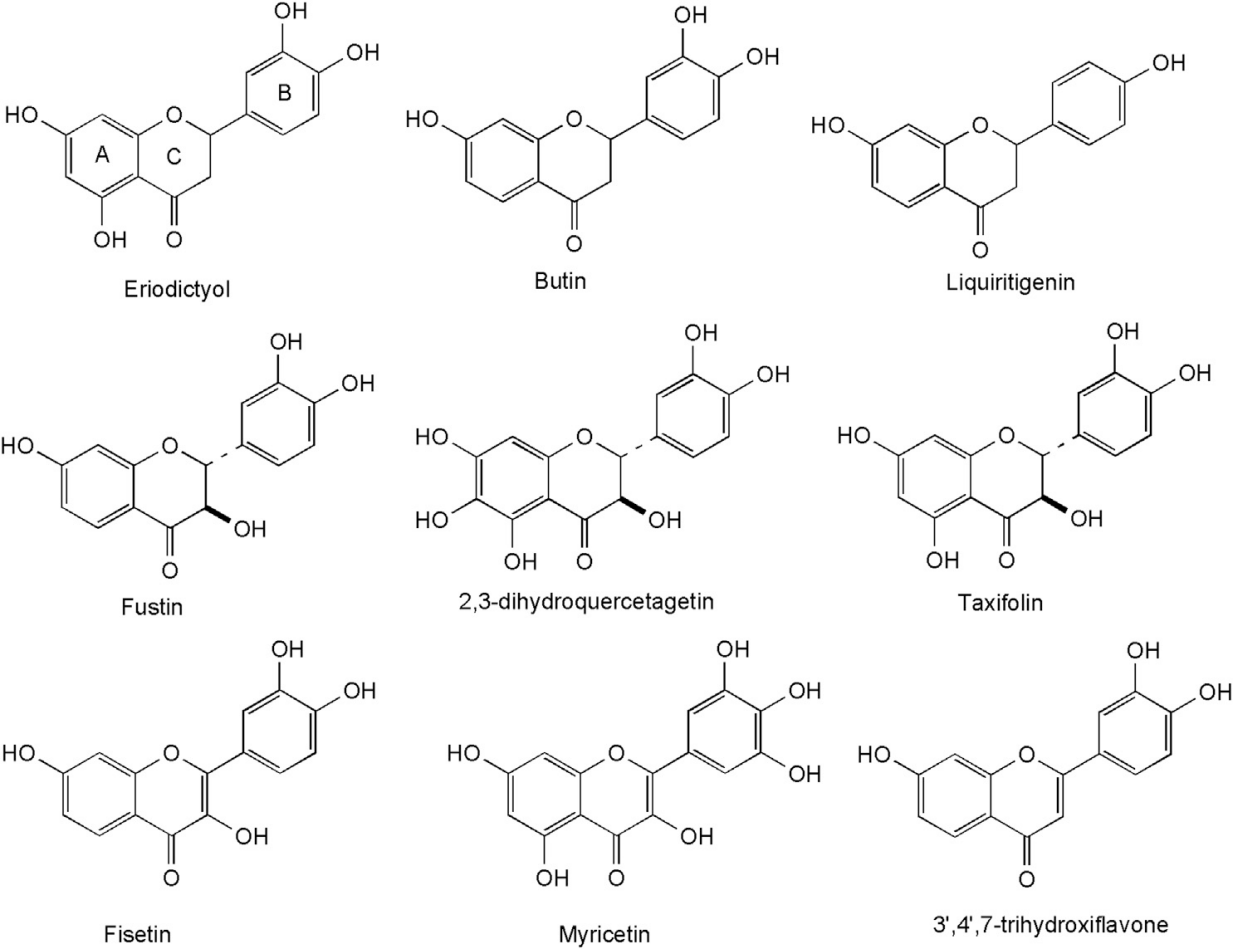
Sulfuretin and its derivatives–typical aurones in *C. coggygria* wood.

A high number of flavonoids have been isolated and identified in methanol extracts of the heartwood (Figure 3). They include the flavanones: eriodictyol, butin and its dimer (3,3″-butindimer) (Antal et al., 2010), liquiritigenin (4′,7-dihydroxyflavanone) and 4′,5,7-trihydroxyflavanone (Valianou et al., 2009), 3′,5,5′,7-tetrahydroxyflavanone (Novaković et al., 2019), the flavanonols: taxifolin and 4′,7-dihydroxyflavanol (Valianou et al., 2009), 2,3-dihydroquercetagetin (Antal et al., 2010), 2,3-trans-fustin, 3-O-methyl-2,3-trans-fustin, 3-O-galloyl-2,3-trans-fustin (Novaković et al., 2019), the flavonols: fisetin, quercetin (Antal et al., 2010) and myricetin (Matić et al., 2013), as well as the flavone: 3′,4′,7- trihydroxyflavone (Novaković et al., 2019). A hallmark of most compounds is the presence of a hydroxy group in position 7 and the absence of a hydroxy-group in position 5 of the A-ring, considered as taxonomical markers for *C. coggygria* (Novaković et al., 2019).

**FIGURE 4:**
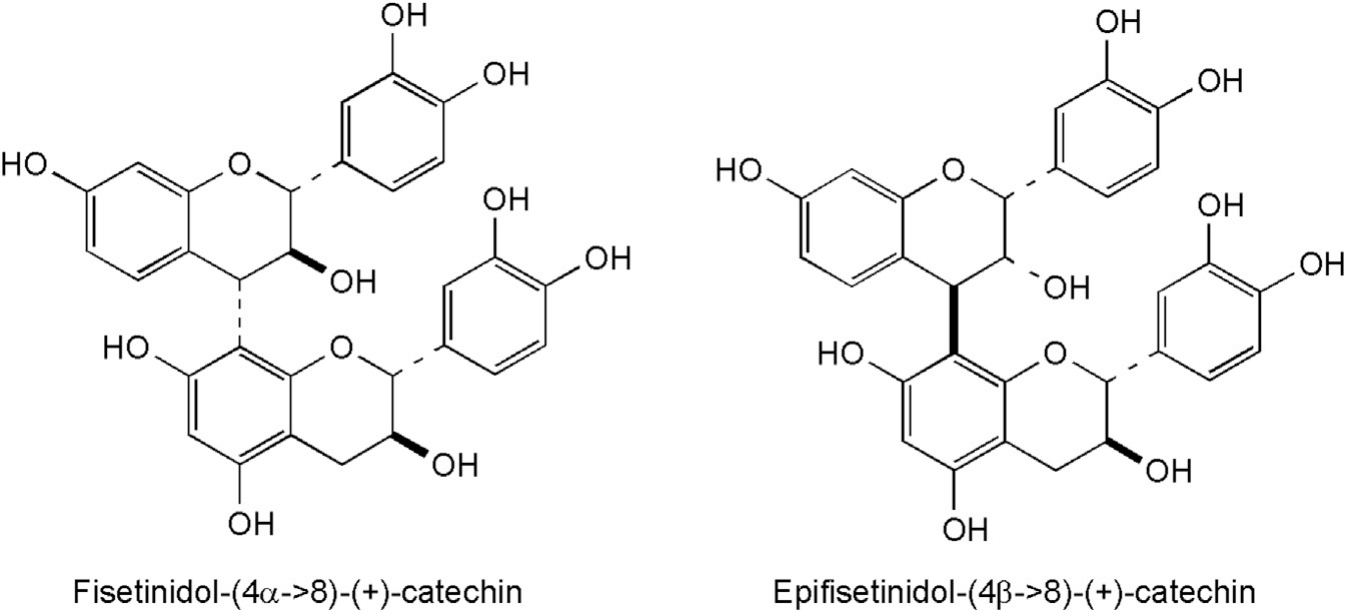
Representative flavonoids with a 6-membered C-ring from the heartwood of *C. coggygria*, consistently displaying a hydroxy group in position 7 of the A-ring.

Several derivatives of flavan-3-ols were isolated and structurally identified in the heartwood (Antal et al., 2010). These plant phenolics are frequent in plants, but particular attention has been given to their occurrence and biologic activity in green tea, pine bark, cacao beans, grape, cranberries and hawthorn (de la Luz Cádiz-Gurrea et al., 2014). The occurrence of polyphenolic dimers is typical for Anacardiaceae plants^2^[Stevens APG website]. Two proanthocyanidins (fisetinidol-(4α→8)-(+)-catechin and epifisetinidol-(4β→8)-(+)-catechin, Figure 4) were reported so far from smoketree (Antal et al., 2010) They were obtained from the diethyl-ether soluble phase of *C. coggygria* crude extract, following a four-step procedure (vacuum liquid chromatography on RP-18 material, followed by gel-filtration on Sephadex LH-20, high-speed coutercurrent chromatogrphy, and a second column chromatography using Sephadex LH-20). Their structure assignment of proanthocyanidins presented a challenging case of signal duplication due to rotational isomerism (Antal et al., 2010). Dimers occur beside monomer catechin, isolated as a racemic mixture.

Other compounds. A series of phenolic acids were isolated from the heartwood of the plant: gallic acid and its methyl ether in leaves, twigs and heartwood (Westenburg et al., 2000; Antal et al., 2010), galloylshikimic acid, trigallic acid, methyl digallate, trigallate (Rendeková et al., 2016), and β-resorcylic acid (Novaković et al., 2019) in the heartwood. According to Matić and co-workers, the methanol extract of *C. coggygria* stem contains chlorogenic, caffeic, coumaric, ferulic and rosmaric acid, with the latter being the major representative of its class in the extract (Matić et al., 2013). Moreover, the heartwood also contains 3-O-β-sitosterol glucoside (Novaković et al., 2019).

### Young Shoots

Young branches have a different flavonoid profile than the heartwood. The flavonoids here include kaempferol-3-O-glucoside, luteolin-7-O-glucoside, luteolin-8C-glucoside (orientin), and apigenin glycoside. Beside them, gallic acid and its derivatives were as well identified (Marčetić et al., 2013).

### Leaves

As leaves were mainly considered a source of tannins, reports on the structure of leaf flavonoids are scarce. Early studies showed that these compounds are based on three main aglycons: myricetin, quercetin, and kaempferol. Sugars of flavonoid glycosides are bound in position 3 of the aglycon and are represented by D-glucose, L-rhamnose, and L-arabinose. Glycosides of the myricetin group (75–80%) are dominant over those of the quercetin group (18–23%), while kampferol glycosides are present in traces (Buziashvili et al., 1973). These early results were confirmed by recent HPLC analysis, identifying the glucosides and rhamnosides of myricetin and quercetin in crude extracts of smoketree leaves (Rendeková et al., 2016). Myricetin-3-O-rhamnoside and myricetin-3-O-galactoside were confirmed spectroscopically in the ethylacetat fraction of a leaf extract (Özbek et al., 2016). Another group of flavonoids, the anthocyanins, are responsible for the various shades of red of smoketree leaves. They include delphinidin 3-galactoside and 7-glucoside, cyanidin 3-galactoside (idaein) and 3-glucoside-7-rhamnoside as well as petunidin 3-glucoside (Tanchev and Timberlake, 1969; Iwashina, 1996).

### Essential Oil Components

The volatile organic compounds from *C. coggygria* have thoroughly been investigated in plants from a variety of regions, ranging from Western Europe to the Himalayas. Due to its aromatic scent, the essential oil of the plant is used in perfumery. Bulgaria is one of the countries producing essential oil form leaves and young twigs of smoketree (Tsankova et al., 1993). The main volatile components are monoterpenes. Limonene dominates in plants from Serbia (Novaković et al., 2007) and Italy (Fraternale and Ricci, 2014). In Greek smoketree, the main components varied according to the site, with limonene being dominant in some samples and myrcene in others (Tzakou et al., 2005). Site-specific variations have as well been pointed out in Turkey, where limonene (Demirici et al., 2003), alpha-pinene (Ulukanli et al., 2014) or geranyl acetate (Bahadirli, 2020) were be the main components. Alfa-pinene is the main compound in essential oil from Bulgarian smoketree samples (Tsankova et al., 1993). Smoketree growing in the northern part of India has myrcene as main volatile constituent (Thapa et al., 2020).

## Pharmacological Data on the Smoketree

The long-lasting and consistent use of *C. coggygria* in traditional medicine across countries offers a sound base for its therapeutic use. However, data on the pharmacology of *C. coggygria* extracts are, for now, at a rather basic stage. Among explored properties, the anti-inflammatory effect was the first to attract the attention of researchers and to be tested experimentally (Bezruk and Lyubetskaya, 1969). Extracts obtained from both leaves and heartwood reduced inflammation in rodent models, while the water extract inhibited cyclooxygenases -1 and -2 (Table 1).

**TABLE 1:** Studies evaluating the anti-inflammatory effect of smoketree extracts and fractions.

Extract	Experimental model	Results	References
Water extract of erial parts	*In vitro* inhibition of ovine COX-1 and human recombinant COX-2	EC_50_ = 2.21 ± 0.18 mg/ml for COX-1 inhibition; EC50 = 4.10 ± 0.27 mg/ml for COX-2 inhibition; Extract had best results in comparison to that of other 8 medicinal plants	Ozsoy et al. (2017)
Heartwood/dietly ether fraction of methanol extract standardized to its content in S, B, fustin, 2,3-dihydroquercetagetin, and quercetin	Mouse ear edema (SKH1 male mice) induced with 12-O-tetradecanoyl phorbol-13-acetate; corneometric assessment	External application of 2 mg extract reduced inflammation with 50%; reduction with 26% of the skin dehydration induced by 12-O-tetradecanoyl phorbol-13-acetate	Antal et al. (2015)
Aqueous infusion from *Cotinus coggygria* leaves	Rats with the carrageenan induced paw edema	Protective effect against inflammation following the intragastric administration of the extract (10 ml/kg) for 15 days	Pavlov et al. (2014)
Young shoots/ethyl acetate fraction of acetone extract	Rats with the carrageenan induced paw edema	Doses of 50 mg/kg and 100 mg/kg reduced theoedema with 46.5 ± 18.5% and 76.7 ± 0.0%, respectively	Marčetić et al. (2013)
Total flavonoids isolated from *C. coggygria* leaves	Formalin-induced edema in mice	Oral administration of 80–160 mg/kg 2 h before and 5 h after formalin application reduced edema, decreased proliferation of cellular elements and raised capillary resistance	Bezruk and Lyubetskaya (1969)

COX: cyclooxygenase; EC50: half maximal effective concentration.

### Wound-Healing Effect

In virtually all countries where smoketree is used in traditional phytotherapy, the wound-healing effect is the most cited one. In order to validate this effect (Aksoy et al., 2016), employed a model of excision wounds in rats with experimentally induced diabetes. The topical application of an ointment containing 5% ethanol extract from leaves demonstrated a significant wound healing effect, confirmed histologically. The elevation of hydroxyproline levels, a precursor of collagen, together with favorable antioxidative effects (elevation in glutathione and decrease in malondialdehyde levels) supported the favorable effect of the extract on wound healing. The wound healing effect is also supported by the antimicrobial effect of *C. coggygria* extracts and essential oils, comprehensively reviewed by (Matić et al., 2016). In order to seek scientific validation for the use of smoketree in the treatment of gastritis, Pavlov and co-workers evaluated the benefits of an aqueous infusion from leaves in indomethacin-induced damage of the gastric mucosa. The intra-gastric administration of 10 ml 2% infusion reduced the number and area of ulcerations, as well as their depth and severity. This effect was accompanied by the decrease of malondialdehyde and uric acid levels, reducing as well the activity of alkaline phosphatase (Pavlov et al., 2013a).

### Hepatic Disorders

Traditionally, smoketree preparations were as well used in liver disease (Shen et al., 1991). The hepatoprotective effect were demonstrated in rodent models (Matić et al., 2011; Matić et al., 2013). Liver damage was induced by pyrogallol, and the protective effect of a relatively high dose of methanolic extract (500 mg/kg) from smoketree stem was administered either 2 or 12 h prior to the administration of pyrogallol. The effects were compared to those of myricetin, found to be the main constituent of the extract. Pretreatment with the extract, but as well with myricetin resulted in a hepatoprotective effect, reducing the elevation of serum AST, ALT, ALP and total bilirubin levels that were induced by pyrogallol. Administration of natural compounds was most efficacious when performed 12 h before pyrogallol. In quest of the mechanism of action for the observed effect, the authors explored the status of Akt or protein kinase B in the liver of rats pretreated with smoketree extract or myricetin by immunoblot analysis. Administration of the natural products, either 2 h or 12 h before the pyrogallol application increased Akt activity and phosphorylation. Moreover, an enhanced STAT3 phosphorylation was reported, enabling the adequate activation of the JAK–STAT signaling pathway, an important cascade involved in signal transduction during hepatic injury (Matić et al., 2013). Administration of *C. coggygria* extract lead to an augmentation of acute phase reactants haptoglobin and a2-macroglobulin with protective role in hepatic lesions. It improved as well markers of oxidative stress, and inhibited NF-kB (Matić et al., 2011). The hepatoprotective effect of smoketree is currently used clinically as part of a compound formula used orally in Traditional Chinese Medicine, where it is mixed with other plant extracts obtained from *Schisandra chinesis*, Chuipencao (*Sedum sarmentosum*), wolfberry (*Lycium chinense*), jujube (*Ziziphus jujuba*), gardenia (*Gardenia jasminoides*) (Hao et al., 2018; Su et al., 2018).

Pavlov et al. (2013b) investigated the toxicity of the aqueous infusion prepared from leaves (1, 2, 4%), administered intragastrically for 30 days in a concentration of 10 ml/kg. The authors did not detect pathological modifications in the organs of the treated rats, no subchronic hepatic or renal toxicity were noted. Moreover, no changes in the activity of hepatic enzymes and levels of urea, creatinine, total thyols and triacylglycerols were found. The potential toxicity was further studied for a 20% ethanol infusion; no lesions of the liver and kidney were reported (Ivanova et al., 2013).

### Diabetes

There are some reports of *C. coggygria* form Turkish ethnomedicine, that describe its utilization in diabetes (Kültür, 2007). Some basic steps were performend until now to verify this indication, with regard to the inhibition of key enzymed involved in the disease. A methanol extract and its fractions (petroleum ether, dichloromethane, ethyl acetate, and n-butanol) obtained from leaves smoketree were evaluated *in vitro* with regard to their α-glucosidase and α-amylase inhibitory activities. The ethyl acetate-soluble fraction displayed the highest efficacy (IC_50_ = 0.0082 mg/ml), having an inhibitory activity roughly ten times higher than the other fractions. Alfa-amylase was not inhibited in that experimental setting (Gözcü et al., 2016). The α-glucosidase inhibitory activity was also reported for the ethanol extract prepared from the branches of the shrub. After bioactivity-guided fractionation, 2,3,4,6-penta-*O*-galloyl-β-D-glucose was shown to have the best α-glucosidase inhibitory effect, with an IC_50_ of 0.96 mg/ml. Comparatively, the related compoung with only four gallic acid residues 1,2,3,6-tetra-O-galloyl-β-D-glucose and gallic acid had week inhibitory effects (Cha et al., 2009).

### Cancer

The anti-cancer effects of *C. coggygria* have received the highest attention in the last period, since the last review on smoketree. References to this regard exist in traditional medicine (Kültür, 2007; Redžić, 2007). Some works explored the cytotoxic effect of smoketree extracts and metabolites on different cell lines, while others pointed out protective effects via antioxidant and anti-mutagenic activities (Westenburg et al., 2000; Matić et al., 2013). One of the earliest studies investigating the cytotoxic effects of this plant focused on the methanol extracts from leaves and flowers, rich in gallic acid and its derivatives. The extracts were applied on two human carcinoma cell lines (cervix–HeLa and colon–LS174). The leaf extract was more active on the HeLa cell line (IC_50_ = 19.1 ± 3.9% μg/mL), while the flower extract had a slightly better inhibition of the LS174 cell line (IC_50_ = 41.3 ± 3.9 μg/ml) (Šavikin et al., 2009).

Gospodinova and co-workers investigated the effects of an aqueous ethanolic extract from smoketree leaves, standardized in total polyphenols and flavonoids (2017 a,b,c). The cytotoxicity tests involving human breast cancer cells (MCF7) showed a significant cytotoxic activity (IC_50_ = 40.6 μg/ml) which was not dose-dependent (Gospodinova et al., 2017a). In a second study, the inhibitory effects on three additional cell lines (breast cancer T47D, cervical cancer HeLa, and ovarian cancer A2780) were explored, in comparison a non-cancerous cell line (breast epithelial cell line MCF10A). Ovarian cancer cells were the most sensitive, being inhibited at an IC_50_ = 30.8 μg/ml; a certain degree of selectivity against cancer cells were noted in comparison with normal cells (Gospodinova et al., 2017b). The research of a possible mechanism of action lead to the investigation of histone deacetylase activity in MCF7 cells following the application of the leaf extract; high levels of histone deacetylase activity is characteristic for cancer cells. The authors reported a significant reduction of HDAC5 and HDAC7 mRNA transcripts at 2 days post-treatment. A tendency to reduce HDAC3 transcriptional levels also resulted from the research (Gospodinova et al., 2017c). The last paper of this work-group focused on the cytotoxic effects against A431 cells (human squamous cell carcinoma), in comparison with normal skin cells (BJ line). The selectivity against cancer cells was observed, and the best anticancer activity was achieved by the chloroformic fraction of the extract, in comparison to that of the aqueous fraction (Gospodinova et al., 2020). Another extract obtained from leaves with ethyl acetate as solvent was applied on normal skin cells (BJ) and mammary gland epithelium cells (MCF-10A). The extract inhibited the proliferation of activated human fibroblasts expressing fibroblast activation protein α (FAP). This is a serine protease implicated in the genesis and progression of tumors (Iliev et al., 2020). In the study of (Pollio et al., 2016), ethanol extract of *C. coggygria* branches and leaves showed significant cytotoxic effects, and both cell morphology and growth were affected. The effects on the cell cycle were consistent in all cell lines (A549, MCF7, TK6 and U937), irrespective of their phenotype (adherent or in suspension). The expression profiles of distinct proteins controlling the progression of the cell cycle and cell death were altered. The percentage of cells in the G1 phase was induced by the extract (Pollio et al., 2016).

The cytotoxic effect of *C. coggygria* on gliobastoma cells (lines U87, U251, and DBTRG-05MG) were investigated by (Wang et al., 2015a; Wang et al., 2015b; Wang et al., 2016). The authors evaluated an ethanol (95%) extract from roots and stems, which contained 4% flavonoids: rutin, fisetin, quercetin, and myricetin. These flavonoids were also used as controls in the assays. The extract was able to inhibit the proliferation of the three cell lines (IC_50_ = 128.49 μg/ml for U87, 107.62 μg/ml for U251, and 93.57 μg/ml for DBTRG-05MG). It induced apoptosis via Akt inhibition, coupled with ERK protein expression. Moreover, the researchers evaluated the significance of their findings in a xenograft model glioblastoma multiforme in female BALB/c mice, reporting a significant antitumor effect. The reduction of the tumor volume after administration of 25 and 50 mg/kg extract (evaluated at 7, 14, 21, and 28 days) was in the same range as in case of the positive control, temozolomide (Wang et al., 2015a). In a subsequent study, the same extract was loaded into polyvinylpyrrolidone K-30/sodium dodecyl sulfate and polyethyleneglycol-coated liposome, in order to augment its solubility and to achieve an improved cerebral targeting. The cytotoxic mechanism of action of this preparation was studied *in vitro*. A DBTRG-05MG glioblastoma cell line was employed. Results pointed to a caspase-dependent activation of both the intrinsic and extrinsic signaling pathways of apoptosis. The proposed mechanism of the proapoptotic effect in glioblastoma cells involved the blockade of the SIRT1/p53-mediated mitochondrial pathway and of the Akt pathway (Wang et al., 2015b). The significant inhibitory effect of glioblastoma cells that the ethanol extract from roots/stem of *C. coggygria* had, was confirmed by an additional work. This study was also performed on a second extract (total flavonoids isolated from *C. coggygria* var. *cinerea*). Both extracts reduced cell proliferation and downregulated the signaling pathways PI3K/Akt and ERK (Wang et al., 2016).

The cytotoxic effects of the diethyl ether-soluble fraction obtained from smoketree heartwood were assessed before and after complexation with two cyclodextrins. Along with the extract, two of its marker compounds (the aurone sulfuretin and the chalcone butein) were as well studied. Among the four cell lines that were used in the work (HeLa, A2780, MCF7, and MDA-MB-231), the most sensitive against *C. coggygria* extract was ovarian carcinoma cell line A2780. The proliferation of these cells were inhibited in the low micromolar range. Butein had the highest activity against HeLa cells, both included in randomly methylated-β-cyclodextrin and in uncomplexed form. Sulfuretin and its complexes had a generally weaker cytotoxicity (Antal et al., 2016).

### Other Effects

As for many other medicinal plants, in addition to the validation of effects known from traditional medicine, modern research aims to expand the uses of this species. In this regard, a potential field is represented by neurological disorders. Eftimov and co-workers explored the effects of an aqueous infusion from leaves (1, 2 and 4%, 10 ml/kg) on the behavior of rats, and its protective effect against lipid peroxidation (Eftimov et al., 2016). The effects were evaluated after 30 days of treatment. In the forced swim test, the timespan of immobility increased, and the effect was significant for the 2% infusion. The infusion had no sedative effect, did not induce motor dis-coordination, but reduced signs of depression. The decrease in malondialdehyde levels was not significant and the authors concluded that the positive effects on brain function were not related to an antioxidative effect (Eftimov et al., 2016).

Acetylcholine esterase inhibition is a major target for drugs combating neurodegenerative conditions like Alzheimer’s disease and Parkinson’s disease. A screening of various plant extracts with Ellman’s reagent pointed to *C. coggygria* as having a real potential to inhibit this enzyme. The best activity was achieved by the crude methanol extract of the heartwood (IC_50_ = 89.3 μg/ml). In order to find the most active compounds, this extract was partitioned in solvents of increasing polarities (petroleum ether, diethyl ether, ethyl acetate, n-butanol, and water), with the diethyl ether fraction having the highest efficacy (IC_50_ = 25.4 μg/ml) due to its content in sulfuretin (IC_50_ = 29.9 μM). After intracerebral injection, the extract elicited an increased acetlycoline concentration (Antal et al., 2008).

Recently, *C. coggygria* extracts have been studied with regard to their potential in skin care, evaluating the effects as inhibitors of collagenase and elastase, fundamental components of the connective tissue which are responsible for the resistance and elasticity of the skin. The research also tested tyrosinase inhibitory effect, in order to identify a potential utilization as skin lightener. Departing from ethanol extracts of pedicels and leaves, an activity-guided fractionation was performed in order to point out the most active compounds. After partition, it was the ethyl-acetate soluble fraction of the pedicels that had the best effect on collagenase inhibition accompanied by a moderate inhibition of elastase and tyrosinase. The main components of the active fractions were methyl gallate, astragalin, isoquercetin, and hyperoside (Deniz et al., 2020).

## *Cotinus coggygria and Toxicodendron vernicifluum*: Common Ground

Despite the research performed up to the present on *C. coggygria*, mandatory data are yet missing in the pharmacology of this species, including pharmacokinetic tests and clinical trials. Interestingly, as these data are lacking for extracts and fractions, they exist for some of the main compounds of smoketree heartwood: sulfuretin, fisetin, butein, owing mostly to the presence of these and other compounds in the Chinese lacquer (*Toxicodendron vernicifluum*, syn. *Rhus verniciflua*). In fact, *T. vernicifluum* is has a firmly established position in traditional Asian medicine. Not only have there been performed comprehensive *in vitro* and *in vivo* studies on this species, but clinical data are also available (Kim et al., 2014). Most of these studies used extracts standardized in fustin (>13.0%), fisetin (7.0%), sulfuretin, butein and other compounds (Lee et al., 2009; Lee et al., 2011). The pharmacokinetic profile of several active compounds has also been reported (Jin et al., 2015).

For *T. vernicifluum*, over 175 constituents have been isolated and described. A review of the literature on key compounds from smoketree heartwood shows that *C. coggygria* and *T. vernicifluum* share an impressive pool of secondary metabolites (Table 2). This situation may even change the systematics of *C. coggygria* in reassigning it back to its initial genus, *Rhus* (Novaković et al., 2019). In both species, the major components are sulfuretin and fustin (Antal et al., 2010; Li M. C. et al., 2020).

**TABLE 2:** Secondary metabolites occuring both in the heartwoods of *Cotinus coggygria* (syn. *Rhus cotinus*) and *Toxicodendron vernicifluum* (syn. *Rhus verniciflua*).

Compound	*Cotinus coggygria*	*Toxicodendron vernicifluum*
Sulfuretin	Westenburg et al. (2000)	Li Y. et al. (2020)
Butein	Valianou et al. (2009)	Li M. C. et al. (2020)
Isoliquiritigenin (*trans*-2′,4.4′-trihydroxychalcone)	Valianou et al. (2009)	Li W. et al. (2020)
Eriodictyol	Antal et al. (2010)	Li Y. et al. (2020)
Butin	Antal et al. (2010)	Li M. C. et al. (2020)
3.3″-butindimer	Antal et al. (2010)	Li W. et al. (2020)
Liquiritigenin (4′,7-dihydroxyflavanone)	Valianou et al. (2009)	Li Y. et al. (2020)
Taxifolin	Valianou et al. (2009)	Jin et al., 2015
2,3-Dihydroquercetagetin	Antal et al. (2010)	Li M. C. et al. (2020)
2,3-trans-Fustin	Novaković et al. (2019)	Li W. et al. (2020)
3-*O*-methyl-2,3-*trans*-fustin	Novaković et al. (2019)	—
3-*O*-galloyl-2,3-*trans*-fustin	Novaković et al. (2019)	Li Y. et al. (2020)
Fisetin	Antal et al. (2010)	Li M. C. et al. (2020)
Quercetin	Antal et al. (2010)	Hashida et al., 2014
3′,4′,7- trihydroxyflavone	Novaković et al. (2019)	Kim et al., 2010
Gallic acid	Antal et al. (2010)	Kim et al., 2010
Methyl gallate	Antal et al. (2010)	Yang P.Y. et al. (2018)
β-resorcylic acid	Novaković et al. (2019)	Li W. et al. (2020)

The high number of shared compounds points to possible similarities in the pharmacology of both species. Moreover, it may provide a significant support in advancing the practical use of the European species into other fields of therapeutic significance, which have already been well developed for *T. vernicifluum*. To this date, the pharmacologic activities that have been reported for this species are: anti-inflammatory, anti-oxidant, anti-cancer, anti-microbial, anti-diabetic, anti-dislipidemic, anti-platlet, anti-vasoconstrictor, as well as protective of the liver, kidneys, neurons and gastro-intestinal tract. The numerous *in vitro* and *in vivo* highlighting these effects make the object of a well-documented review by (Li W. et al., 2020). A significant drawback of scientific literature focused on the pharmacology of plant extracts, including those from *T. vernicifluum*, is represented by missing or incomplete data on the plant part, the extraction solvent(s) and key marker compounds. Representative studies investigating lacquer extracts standardized in compounds that occur in both species are listed in Table 2. An important advantage of *C. coggygria* over *T. vernicifluum* is the absence of allergenic urushiols (Ippen 1983; Bertrand et al., 2008), which are common in the Asian species (Li et al., 2021).

## Bioactivity of Common Secondary Metabolites

**FIGURE 5:**
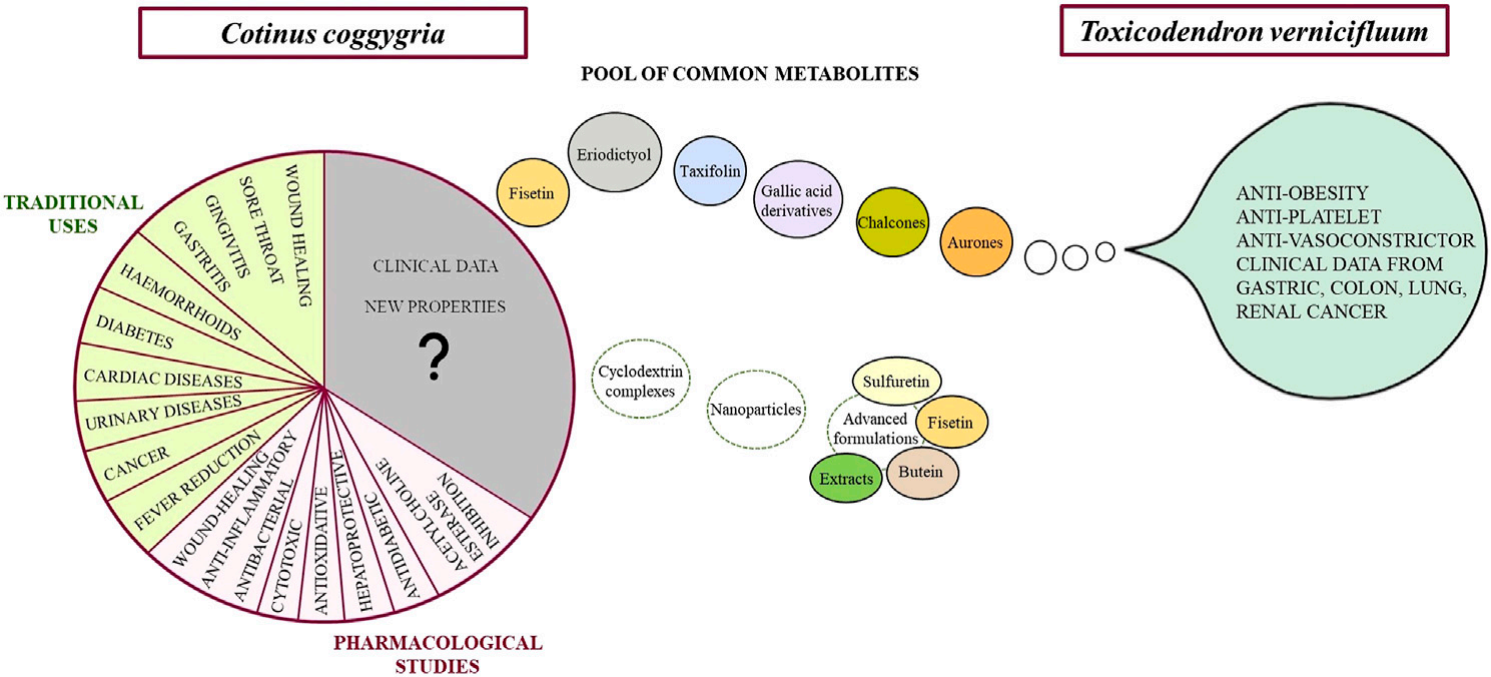
Benefits of an interconnected approach for an optimized valorization of smoketree.

**FIGURE 6:**
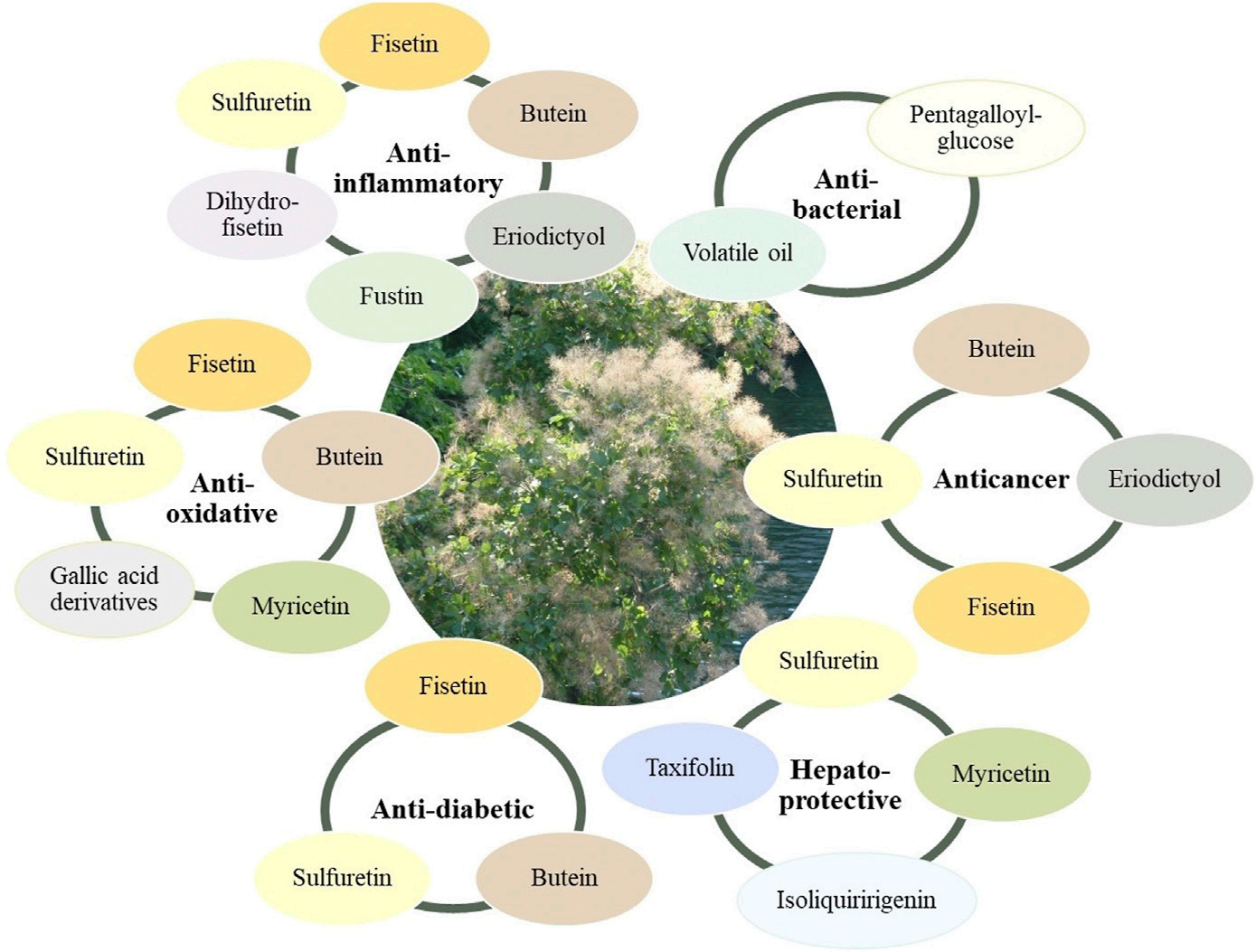
Metabolites from smoketree that have been reported to be responsible for the main pharmacological effects of smoketree.

Key elements in understanding and advancing the pharmacology of smoketree come from studies investigating the metabolites that occur in the plant. Most of the available data come from research on fisetin, sulfuretin and butein, important flavonoids occuring in the woody parts of *C. coggygria* and *T. vernicifluum* (Figure 5,6).

Fisetin benefited from a special attention in recent research (Chen et al., 2020; Yan et al., 2021). This flavonol is also present in many edible plants such as strawberries, onions, apples or persimmons. The fisetin content in plants is between 2 and 160 μg/g (Kashyap et al., 2018). It was first isolated from *Rhus cotinus* and studies conducted over time showed anti-inflammatory, anticancer, antioxidant and neuroprotective effects (Grynkiewicz and Demchuk, 2019). Of particular interest is ability of fisetin to reduce senescence markers in mice and human tissues. A reduction of senescent cells fraction in white adipose tissue of naturally aged C57BL/6 mice after fisetin treatment was reported (Yousefzadeh et al., 2018). Evaluation of fisetin effects in bleomycin-induced pulmonary fibrosis revealed an inhibition of alveolar epithelium cell senescence through regulation of AMPK/NF-κB signaling pathway. The anti-fibrotic activity of fisetin was therefore associated with the senolytic properties of this compound (Zhang et al., 2020). The accumulation of senescent cells has been associated with aging and with age-related dysfunctions. The characteristics of these cells have been studied and agents capable of eliminating them have been sought (Wissler Gerdes et al., 2020). Thus, senolytic agents, including fisetin, have been proposed to delay aging and reduce the severity of age-related diseases (Yousefzadeh et al., 2018).

Fisetin exerts an antiatherosclerotic effect in mice by reducing the lipid and malondialdehyde (MDA) levels and increasing the level of superoxide dismutase (SOD). The reduction of the atherosclerotic plaque was observed. Fisetin also regulates the expression of LOX-1 (lectin-like oxidized low-density lipoprotein receptor-1) and PCSK9 (proprotein convertase subtilisin/kexin type 9) thus improving lipid metabolism (Yan et al., 2021). Association of fisetin with recombinant tissue plasminogen activator extended the therapeutic window of treatment in acute ischemic stroke. Patients receiving 100 mg fisetin daily for seven days exhibited lower levels of C-reactive protein, MMP-2 (matrix metalloproteinase-2) and MMP-9 (matrix metalloproteinase-9). Therefore, fisetin has shown potential for use as a supplement in reperfusion therapy with recombinant tissue plasminogen activator (Wang et al., 2019). A protective effect on ischemic cardiomyocytes was observed when fisetin was tested in a rat ischemia/reperfusion injury model. Fisetin reduced apoptosis in myocardial cells, prolonged coagulation and decreased Von Willebrand factor plasma levels (Long et al., 2020).

Given the association of neurodegenerative diseases with alterations in gut microbiota, the effects of fisetin as neuroprotective agent have been studied in a mice model of Parkinson’s disease. Fisetin improved motor behavior and protected against dopaminergic neurodegeneration induced by MPTP (1-methyl-4-phenyl-1,2,3,6-tetrahydropyridine). It also increased the abundance of Lachnospiraceae, activity associated with its neuroprotective properties (Chen et al., 2020).

The anticancer effect of fisetin has also been studied, and the mechanisms by which it acts in cancer have been highlighted in several studies. Its effects have been evaluated in prostate cancer, colon cancer, pancreatic cancer, liver cancer, lung cancer, ovarian cancer or breast cancer cell lines (Imran et al., 2020). Fisetin inhibits the proliferation of pancreatic cancer cells PANC-1 and induces autophagy and apoptosis (Jia et al., 2019). It has been proposed as an adjuvant to 5-fluorouracil treatment in PIK3CA-mutant colorectal cancer. The association of 5-fluorouracil and fisetin resulted in a marked decrease in the viability of cancer cells. The combination determined inhibition of AKT phosphorylation and decreased PI3K (phosphatidylinositide-3-kinase) expression. A preventive effect against tumor formation was also observed for fisetin (Khan et al., 2019). Fisetin acts on oral squamous cell carcinoma by targeting PAK4 (p21-activated kinase 4). Promotion of cell apoptosis and inhibition of proliferation and migration have been reported after treatment of cancer cells with fisetin (Li W. et al., 2020). A clinical trial that included patients with colorectal cancer evaluated the effects of fisetin on inflammatory status. Administration of 100 mg fisetin per day for seven weeks, before and during chemotherapy, resulted in a decrease of hs-CRP (high-sensitivity C-reactive protein) and IL-8 (interleukin-8) levels in the intervention group, revealing the anti-inflammatory activity of this compound when used as a complementary treatment in colorectal cancer (Farsad-Naeimi et al., 2018).

Fisetin has also been studied as an anti-diabetic. In an *in vitro* model of experimental diabetes, oral administration of fisetin (10 mg/kg) reduced blood glucose and glycosylated hemoglobin levels, while the plasma insulin level was increased. This flavonoid also lead to a decrease of mRNA and expression levels of gluconeogenic genes (phosphoenolpyruvate carboxykinase, glucose-6-phosphatase) (Prasath et al., 2014). The favorable effects of gluconeogenesis inhibition in the liver by fisetin are of relevance for the antidiabetic effects of both *C. coggygria* and *T. vernicifluum*.

A recent study showed that fisetin stimulates hair growth. When applied topically in C57BL/6 mice, fisetin acts on hair growth by stimulating the transition from telogen to anagen phase, effect related to its activity on TERT (telomerase reverse transcriptase) expression in the dorsal skin cells of treated mice (Kubo et al., 2020).

Sulfuretin is a major compoud of smoketree wood, emblematic for its golden color. Among the reported effects for this compound are the anti-inflammatory, anticancer, neuroprotective and antidiabetic ones (Pariyar et al., 2017). One of the first studies reported that this aurone is active in rheumatoid arthritis (Choi et al., 2003). The research of the mechanism of the anti-inflammatory pointed to an induction of heme oxygenase-1 expression (Lee et al., 2010), inhibition of LPS-induced inducible nitric oxide synthase, inhibition of cyclooxygenase-2, and reduction of the expression of pro-inflammatory cytokines via down-regulation of NF-kappaB (Shin et al., 2010). *In vivo.* sulfuretin maintained joint integrity, a result underscored by radiologic and histopathologic evidence (Lee et al., 2012). As a potential anti-diabetic, sulfuretin inhibits aldose reductase, diminishes formation of glycation end products (Lee et al., 2008), and protects against cytokine-induced beta-cell damage in experimentally induced diabetes (Song et al., 2010). The anti-obesity effect of sulfuretin was emphasized in a study performed on obese mice. Decrease of total cholesterol and triglycerides levels, inhibition of lipid accumulation, as well as improvement of glucose metabolism were observed. Sulfuretin also prevented body weight gain (Kim et al., 2019). The anti-adipogenic activity has been associated with the properties of sulfuretin to supress adipogenic factors such as PPARγ (peroxisome proliferator-activated receptor γ), C/EBPα (CCAAT/enhancer-binding protein α) and C/EBPβ. Sulfuretin exhibited dose and time dependent proliferation-reducing effects of 3T3-L1 preadipocytes cells (Lamichhane et al., 2017).

Hepatoprotective properties have as well been reported for sulfuretin. When tested on L02 hepatic cell line, sulfuretin decreased reactive oxygen species levels and protected cells against treatment with palmitate. It acts by promoting mitophagy and the phenolic hydroxyl group was considered to be essential for the cytoprotective properties (Lu et al., 2019).

Sulfuretin showed beneficial effects in an atopic dermatitis mouse model, inhibiting IL4 production and improving several symptoms of the disease. It reduced the severity of skin lesions, the scratching frequency and decreased IgE serum levels (Jiang and Sun, 2018).

Anti-Parkinson activity was evaluated in SH-SY5Y neuroblastoma cells, revealing a protective effect of sulfuretin against the cytotoxicity induced by MPP^+^ (1-methyl-4-phenyl pyridinium) neurotoxin. The neuroprotective effects are due to the effects of sulfuretin on ERK and Akt/GSK3β pathways (Pariyar et al., 2017).

Butein. Research regarding the biological properties of butein highlighted a wide range of effects, that indicated a potential of use in chronic conditions due to anti-inflammatory, antioxidant, antidiabetic, neuroprotective and anticancer effects (Padmavathi et al., 2017). The effects of this compound correlate with the medicinal properties reported in ethnopharmaceutic and pharmacological studies. Its anti-ulcerative activity was evaluated in mice with gastric ulcer induced with indomethacin and the administration of butein (10, 20, 40 mg/kg) reduced inflammatory parameters, increased PGE2 (prostaglandin E2) concentrations and reduced ulcer areas (Ugan and Un, 2020). Butein exerts preventive effects against functional β-cell damage, slowing the progression of type 1 diabetes mellitus. This effect was studied in cytokine-induced β-cell damage. The chalcone blocked cytokine-induced NO production, the expression of iNOS, the translocation of NF-κB and inhibited insulin secretion stimulation by glucose (Jeong et al., 2011). Butein has a significant anti-inflammatory effect, inhibiting the production of pro-inflammatory cytokines, matrix metalloproteinases, expression of enzymes involved in inflammation such as COX-2; in experimentally induced osteoarthritis it has been shown to reduce cartilage erosion and synovitis (Zheng et al., 2017). Several studies have evaluated the cytotoxic effects of butein on cell lines including human cervical cancer (C-33A, SiHa) (Yang J. et al., 2018), breast cancer MCF7 and T47D (Huang et al., 2020), non-small-cell lung cancer (Di et al., 2019), human oral squamous cancer (SAS, KB) (Bordoloi et al., 2019) and many other. The compound induces apoptosis by activating the Bax-caspase-3-PARP pathway, it induces the arrest of the cell cycle at the G2/M transition, blocks telomerase activity, reduces angiogenesis and has anti-metastatic properties (Jayasooriya et al., 2018).

The health-promoting effects of smoketree extracts are further supported by pharmacological data other secondary metabolites, including fustin, eriodictyol, isoliquiritigenin, taxifolin, myricetin and pentagalloyl-glucose. Fustin (dihydrofisetin), a major flavanonol derivative in heartwoods of *C. coggygria* and *T. vernicifluum*, has antioxidative, antiproliferative and anti-inflammatory effects (Jung et al., 2007). Fustin and an extract of *T. vernicifluum* branches standardized in 3% of this compound were tested in lipopolysaccharide (LPS)-stimulated rats and were able to prevent the elevation of interleukin-6 cytokine, as well as the expression of iNOS, and COX-2 mRNA expression (Moon et al., 2015). This flavonoid also protects against 6-hydroxydopamine-induced neuronal cell death (Park et al., 2007) and reduces experimentally induced learning impairment, due to a muscarinic M1 receptor-mediated anticholinergic effect (Jin et al., 2009).

Eriodictyol is known for anti-inflammatory, cardioprotective, neuroprotective, anti-obesity, antioxidant and anti-cancer (Islam et al., 2020) effects. This flavanone induces apoptosis of CHG-5 and U87MG glioma cell lines, thus presenting anti-tumor effects. The mechanism involved is related to the downregulation of PI3K/Akt/NF-κB signaling pathway (Li et al., 2021). Eriodictyol also targets MEK/ERK signaling pathway, exhibiting antiproliferative properties in human nasopharyngeal CNE1 cancer cells. It inhibits the migration and invasion of cancer cells and induces autophagy (Tang et al., 2020). The anti-arthritic effect of eriodictyol was shown in a study performed in a rat model of rheumatoid arthritis. Eriodictyol (20 mg/kg or 40 mg/kg) was administered orally in rats with collagen-induced rheumatoid arthritis for four weeks and it determined a reduction of paw swelling and decreased IL-6, IL-1β and TNF-α levels (Lei et al., 2020). Eriodictyol showed positive effects on dyslipidemia, insulin resistance, inflammation and hepatic steatosis when administered in diet induced C57BL/6N obese mice. A decrease in triglyceride, total cholesterol and free fatty acids levels was observed after dietary supplementation with 0.005% w/w eriodictyol for 16 weeks. Plasma glucose and plasma levels of pro-inflammatory cytokines were also reduced (Kwon and Choi, 2019).

The chalcone isoliquiritigenin is mostly known from licorice species (*Glycyrrhiza* sp.). Its anti-inflammatory, anti-oxidative, anticancer and protective (hepatoprotective, cardioprotective) effects have been reviewed by (Peng et al., 2015). Taxifolin, myricetin and quercetin are well-studied flavonoids that are already marketed as dietary supplements. They have a widespread occurrence in the Plant Kingdom and their activity has extensively been covered by recent reviews (Andres et al., 2018; Sunil and Xu, 2019; Song et al., 2020).

Pentagalloyl glucose is a hydrolysable tannin with a high antioxidant activity due to the numerous phenolic hydroxy groups in its structure. Its properties include anti-inflammatory, anti-bacterial, antidiabetic, and anti-cancer properties. It is able to inhibit DNA replication, cause cell cyle arrest in the G1 and S phases, and elicit apoptosis (Zhang et al., 2009). This compound stands out due to its ability to bond to proteins, especially those with a high-proline content like collagens, via hydrogen bonds and hydrophobic forces. This property makes pentagalloylglucose a candidate in the treatment of vascular disease (Patnaik et al., 2019). The ability to precipitate bacterial proteins translates into an intense bacteriostatic effect, combating both Gram-positive and Gam-negative bacteria like: methicillin-resistant and quinolone-resistant *Staphylococcus aureus*, *Streptococcus mutans, Escherichia coli,* and *Pseudomonas eruginosa* (Cho et al., 2010).

## Perspectives

*Cotinus coggygria* Scop. is a species brought in to the attention of modern pharmacological research via centuries-old traditional medicine. It is not included in European Pharmacopoeas. Most of the effects mentioned in ethnopharmacy found support after scrutiny with *in vitro* and *in vivo* experimental models: wound-healing, anti-bacterial, anti-inflammatory. The chemical composition of the plant includes three major groups of compounds–tannins, volatile organic compounds and flavonoids. With regard to the latter, smoketree showcases a distinctive profile of 5-deoxyflavonoids consisting in notable amounts of sulfuretin, fisetin and butein. Interestingly, this rare profile is shared by the lacquer tree (*Toxicodendron vernicifluum*), a species of Asian origin that is taxonomically related to smoketree. While biomedical research performed on this well-established species in Asian phytotherapy is superposing with some known health-promoting activities of the smoketree, it has a real potential to guide research and utilization toward new therapies. These chiefly include obesity, diabetes, metabolic syndrome, vascular disease and cancer treatment.

## Footnotes

1 The Plant List, ^ http://www.theplantlist.org/browse/A/Anacardiaceae/Cotinus/ (Accessed January 26, 2021) Plants of the World online, ^ http://www.plantsoftheworldonline.org/ (Accessed January 26, 2021).

2 Stevens, P.F. Angiosperm Phylogeny Website. ^ http://www.mobot.org/MOBOT/research/APweb/ (Accessed December 3, 2020)
